# Prediction of ineffectiveness of biological drugs using machine learning and explainable AI methods: data from the Austrian Biological Registry BioReg

**DOI:** 10.1186/s13075-024-03277-x

**Published:** 2024-02-08

**Authors:** Dubravka Ukalovic, Burkhard F. Leeb, Bernhard Rintelen, Gabriela Eichbauer-Sturm, Peter Spellitz, Rudolf Puchner, Manfred Herold, Miriam Stetter, Vera Ferincz, Johannes Resch-Passini, Jochen Zwerina, Marcus Zimmermann-Rittereiser, Ruth Fritsch-Stork

**Affiliations:** 1 Siemens Healthcare GmbH, Computed Tomography, Forchheim, Germany; 2 Rheumatological Practice, Private Office, Hollabrunn, Austria; 3 Lower Austrian State Hospital Stockerau, 2nd Department of Medicine, Lower Austrian Competence Center for Rheumatology, Karl Landsteiner Institute for Clinical Rheumatology, Stockerau, Austria; 4 Rheumatological Practice, Private Office, Linz, Austria; 5 Rheuma-Center Wien-Oberlaa, Department of Rheumatology, Vienna, Austria; 6 Rheumatological Practice, Private Office, Wels, Austria; 7 Department of Internal Medicine II, Medical University of Innsbruck, Innsbruck, Austria; 8 Rheumatological Practice, Private Office, Amstetten, Austria; 9 Department of Internal Medicine, University Hospital St. Pölten, St. Pölten, Austria; 10 Hanusch Krankenhaus, Vienna, Austria; 11 Ludwig Boltzmann Institute of Osteology, Vienna, Austria; 12 Siemens Healthcare GmbH, Digital & Automation, Erlangen, Germany; 13 Health Care Center Mariahilf of ÖGK, Vienna, Austria; 14 Biologica Registry BioReg, Stockerau, Austria; 15 Medical Faculty, Sigmund Freud Private University Vienna, Vienna, Austria

**Keywords:** Rheumatoid arthritis, bDMARD, Machine learning, Routinely collected data, DMARDs

## Abstract

**Objectives:**

Machine learning models can support an individualized approach in the choice of bDMARDs. We developed prediction models for 5 different bDMARDs using machine learning methods based on patient data derived from the Austrian Biologics Registry (BioReg).

**Methods:**

Data from 1397 patients and 19 variables with at least 100 treat-to-target (t2t) courses per drug were derived from the BioReg biologics registry. Different machine learning algorithms were trained to predict the risk of ineffectiveness for each bDMARD within the first 26 weeks. Cross-validation and hyperparameter optimization were applied to generate the best models. Model quality was assessed by area under the receiver operating characteristic (AUROC). Using explainable AI (XAI), risk-reducing and risk-increasing factors were extracted.

**Results:**

The best models per drug achieved an AUROC score of the following: abatacept, 0.66 (95% CI, 0.54–0.78); adalimumab, 0.70 (95% CI, 0.68–0.74); certolizumab, 0.84 (95% CI, 0.79–0.89); etanercept, 0.68 (95% CI, 0.55–0.87); tocilizumab, 0.72 (95% CI, 0.69–0.77).

The most risk-increasing variables were visual analytic scores (VAS) for abatacept and etanercept and co-therapy with glucocorticoids for adalimumab. Dosage was the most important variable for certolizumab and associated with a lower risk of non-response. Some variables, such as gender and rheumatoid factor (RF), showed opposite impacts depending on the bDMARD.

**Conclusion:**

Ineffectiveness of biological drugs could be predicted with promising accuracy. Interestingly, individual parameters were found to be associated with drug responses in different directions, indicating highly complex interactions. Machine learning can be of help in the decision-process by disentangling these relations.

**Supplementary Information:**

The online version contains supplementary material available at 10.1186/s13075-024-03277-x.

## Introduction

Rheumatoid arthritis (RA) is an autoimmune inflammatory joint disease affecting 0.5–1% of the population worldwide [1]. The last decades have seen great advances in our knowledge of the pathogenesis, which has led to an expanded armamentarium of therapeutical options (and vice versa) [2, 3]. Today’s therapeutical management of RA is governed by several concepts. The paradigm of treating early and using a window of opportunity to prevent joint destruction has become commonly accepted policy [4]. Likewise, a treatment strategy with a clearly defined clinical target is advocated in guidelines internationally under the catchphrase “treat to target” (t2t) [5]. In addition, a patient-tailored approach is pursued in order to forestall unwanted side effects, and respective research is undertaken under the notion of “precision medicine” [6].

Precision medicine is a multilayered system, where certain characteristics stemming from an array of items derived from medical history details to serological or imaging markers to genomic as well as other -omics are chosen to create a model of predicting the clinical response to certain treatments. In this respect, clinical practice favors easily attainable items and gender, disease activity, and duration of symptoms have been identified as parameters distinguishing refractory from treatment amenable rheumatoid arthritis in general [7].

In several reports focusing on the prediction of the response to *specific* disease-modifying drugs (DMARD), genetic biomarkers have surfaced, e.g., the PDE3A–SLCO1C1 locus rs3794271 as marker of a positive response to aTNF-therapy (anti-tumor necrosis factor therapy) [8, 9]. A platform combining the molecular signature of RA patients and clinical data to predict the response to aTNF was introduced in 2021 [10]. Its validity and practicability in academic centers as well as private practices was reported recently proving superiority to the clinical standard guided by recommendations [11]. However, for many practices, this approach may not be feasible due to financial and organizational aspects. Concentrating on readily available patient data, e.g., a predictive role of sex was implied for RA patients on aTNF, favoring male patients in early RA [12].

Machine learning techniques have been used sporadically to predict treatment responses. In this respect, the Korean College of Rheumatology Biologics and Targeted Therapy Registry (KOBIO) was investigated by two studies applying different predictive models for several bDMARDS to predict remission at 1-year follow-up [13, 14] in RA patients as well as patients with spondylarthritis. Lee et al. found random forest method model to have the best prediction performance altogether with AUROC values of 0.638 (95% CI, 0.576–0.658) [13]. An earlier conducted study [14] found AUROC values between 0.511 and 0.694 with Ridge classifier performing the best for one drug (golimumab).

The goal of our study was to develop models to predict the risk of non-response for specific bDMARDs considering a 6-month prediction time window, using solely clinical routine data, and in addition to explain the impact of each clinical feature contributing to the model outcomes.

## Methods

**Fig. 1:**
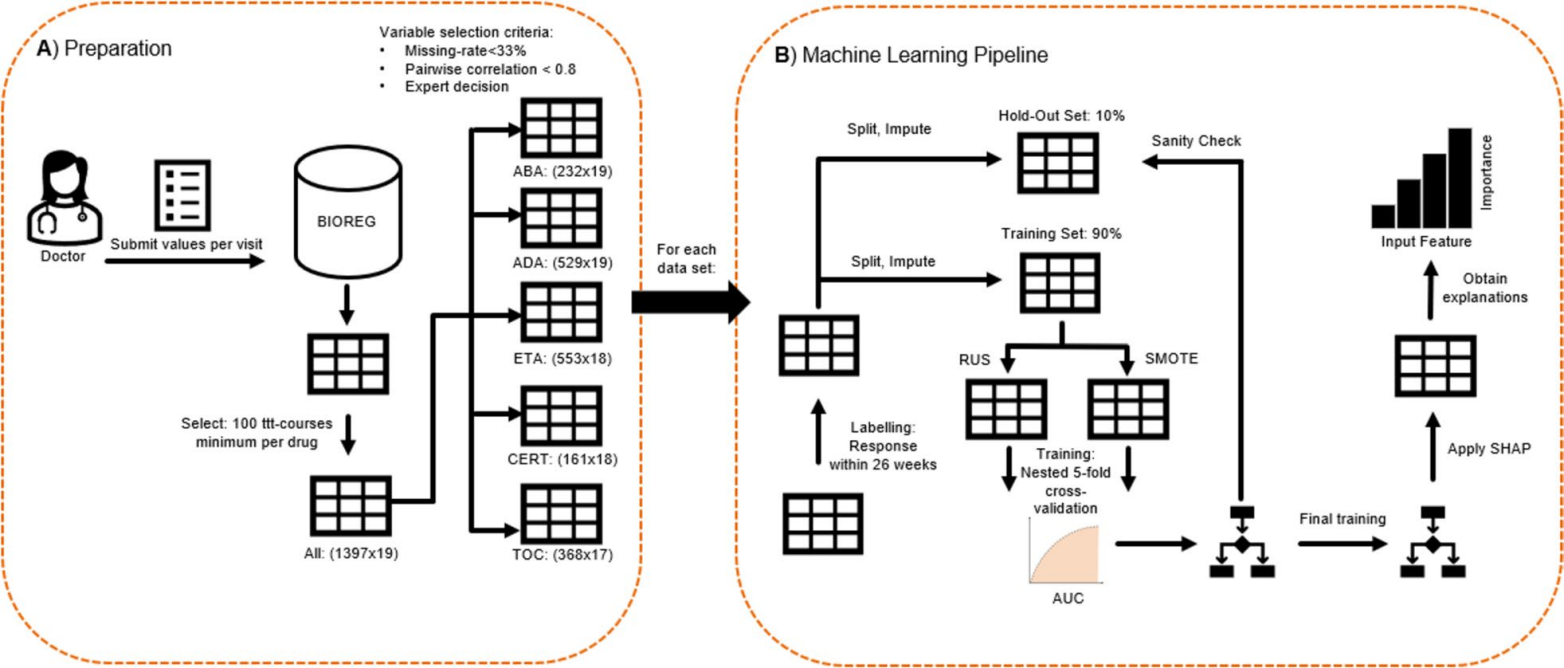
**A** Data preparation. Data were selected based on number of t2t courses. Variables were selected if the missing rate did not exceed 33%. **B** Machine learning pipeline: Data was labeled, depending on the outcome of the therapy course. Iterative imputation was applied, on the hold-out-set (test-set) and on the training set. Sampling strategies were applied, and the AUC (area under the curve) was collected for each model configuration. The final, re-trained model was explained via applying SHAP (SHapley Additive exPlanations)

A high-level overview of the data collection and processing chain is illustrated in Fig. 1 and explained in the following section.

### Patient-derived data

Patient data were obtained from the Austrian Registry for Biologicals, Biosimilars, and targeted synthetic DMARDs in the treatment of inflammatory rheumatic disease—BioReg, which was established in 2010 for the purpose of monitoring those drugs’ safety and efficacy. The registry includes patients suffering from rheumatoid arthritis, psoriatic arthritis, and spondylarthritis [15].

BioReg is a nationwide registry with 8 private rheumatology practices and 12 hospitals spread throughout Austria at the time of the study. Patients with the above mentioned inflammatory rheumatic diseases are included at the start of a new biological treatment. Inclusion criteria of the registry are thus the presence of RA, psoriatic arthritis, or spondylarthritis, age above 18, and the start of a new bDMARD. Exclusion criteria are the presence of other rheumatic diseases and age under 18.

For the present study, data from 1397 patients suffering from RA who were treated with bDMARDs collected from 2010 until 2021 were retrieved. One patient can occur multiple times in the data as the patient can be enrolled to multiple treat-to-target courses. The patient baseline characteristics are presented in Table 1. To obtain markers *predicting* the response, only the baseline visits were considered.

**Table 1:** Characteristics of t2t courses

			**Ineffective**		
		**Overall**	**No**	**Yes**	***P*-Value**
**T2T courses (*n*)**		1843	1724	119	
**BMI, mean (SD), (kg/m**^**2**^**)**		26.4 (4.8)	26.5 (4.8)	26.4 (4.4)	0.830
**Age, mean (SD), year**		56.1 (13.6)	56.0 (13.6)	58.5 (13.5)	0.054
**Gender, *n* (%)**	**M**	407 (22.1)	380 (22.0)	27 (22.7)	0.960
	**F**	1436 (77.9)	1344 (78.0)	92 (77.3)	
**Disease duration, mean (SD), year**		10.4 (8.7)	10.4 (8.7)	11.4 (9.6)	0.257
**IV administration, *n* (%)**	**No**	1610 (87.4)	1507 (87.4)	103 (86.6)	0.897
	**Yes**	233 (12.6)	217 (12.6)	16 (13.4)	
**MTX co-therapy, *n* (%)**	**No**	824 (44.7)	766 (44.4)	58 (48.7)	0.413
	**Yes**	1019 (55.3)	958 (55.6)	61 (51.3)	
**Other DMARD co-therapy, *n* (%)**	**No**	1545 (83.8)	1454 (84.3)	91 (76.5)	**0.033**
	**Yes**	298 (16.2)	270 (15.7)	28 (23.5)	
**GC co-therapy, *n* (%)**	**No**	1156 (62.7)	1105 (64.1)	51 (42.9)	** < 0.001**
	**Yes**	687 (37.3)	619 (35.9)	68 (57.1)	
**Previous aTNF therapy, *n* (%)**	**No**	1233 (66.9)	1168 (67.7)	65 (54.6)	**0.004**
	**Yes**	610 (33.1)	556 (32.3)	54 (45.4)	
**HAQ, mean (SD)**		1.0 (0.7)	1.0 (0.7)	1.2 (0.7)	**0.015**
**Rheuma-factor-positivity, *n* (%)**	**No**	480 (30.0)	443 (29.6)	37 (35.6)	0.241
	**Yes**	1120 (70.0)	1053 (70.4)	67 (64.4)	
**VAS-Pat., mean (SD), mm**		39.6 (24.4)	38.7 (24.2)	51.1 (24.6)	** < 0.001**
**VAS-Ph., mean (SD), mm**		28.7 (20.1)	28.4 (20.1)	32.4 (20.4)	**0.047**
**Anti-CCP, *n* (%)**	**No**	409 (33.2)	380 (32.8)	29 (39.2)	0.317
	**Yes**	823 (66.8)	778 (67.2)	45 (60.8)	
**TJC, mean (SD)**		4.5 (4.7)	4.4 (4.6)	6.0 (5.8)	**0.006**
**SJC, mean (SD)**		3.0 (2.9)	2.9 (2.9)	3.6 (2.8)	**0.020**
**CRP, mean (SD), mg/dL**		8.9 (15.4)	8.6 (15.0)	11.9 (19.8)	0.102
**ESR, mean (SD), mm/h**		19.1 (17.9)	18.9 (17.7)	22.5 (20.8)	0.094
**DAS28-ESR, mean (SD)**		3.8 (1.5)	3.8 (1.5)	4.1 (1.5)	0.101
**Smoker, *n* (%)**	**Current**	161 (8.7)	151 (8.8)	10 (8.4)	0.978
	**Past**	87 (4.7)	81 (4.7)	6 (5.0)	
	**Never**	1595 (86.5)	1492 (86.5)	103 (86.6)	

*aTNF* anti*-*tumor necrosis factor, *CRP* C-reactive protein, *DAS28* Disease Activity Score 28, *ESR* erythrocyte sedimentation rate, *TJC* tender joint count, *HAQ* Health Assessment Questionnaire, *SJC* swollen joint count, *VAS-Pat.* visual analogue scale patient, *VAS-Ph.* visual analogue scale physician, *Anti-CCP* anti-cyclic citrullinated peptide, *MTX* methotrexate, *IV Administration*, intravenous administration, *GC* glucocorticoid

### Exclusion criteria

The originally available raw dataset contained 62 variables for feature generation. We applied several measures to reduce dimensionality, since the datasets per medication were relatively small and to avoid the “curse of dimensionality,” which refers to the problem that more data is often required to represent the variability of a dataset in high-dimensional space. A list of this set of variables, the missing rate, and the reason for exclusion (e.g., missing rate, clinical relevance, correlation higher than 0.8 with other variables or weak association with the outcome label) is presented in the supplemental material in Supplementary Table S1.

After applying the extraction criteria to the raw dataset, the correlation between all variables was assessed and variables were excluded, if the correlation threshold exceeded 0.8.

Due to high correlation of SDAI (Simplified Disease Activity Index) and CDAI (Clinical Disease Activity Index) with tender and swollen joint counts, SDAI and CDAI were excluded to avoid redundancy. The variable encoding the smoking status was excluded due to very weak association with the ineffectiveness of the treatment shown in Table 1.

bDMARDs with less than 100 treat to target (t2t) courses were excluded from the analysis. After obtaining data from the selected bDMARDs, variables were kept, if they reached a completeness rate of at least 67%. This resulted in a slightly different set of variables, depending on the respective bDMARD. After performing the machine learning modeling, an AUC < 0.65 of the models (see below) was set as threshold for further evaluation, since lower AUCs are considered often as poor, weak, or low by medical researchers [16]. Applying those exclusion criteria resulted in a cohort underwent treatment with abatacept, adalimumab, certolizumab, etanercept, or tocilizumab.

### Statistical analysis

After obtaining the cleaned dataset, patient characteristics for the whole cohort were evaluated: Two-sample *t*-test was conducted for numerical variables and chi-squared for categorical variables to assess whether the variables are significantly associated with the outcome of therapy. In addition, the same analysis was applied per medication to evaluate whether similar patterns could be observed after performing the machine learning analysis.

### Machine learning modeling

Predicting non-responders within a t2t course can be translated into a binary classification problem; ineffectiveness was chosen as the independent outcome variable to be predicted, where ineffectiveness was defined by the experience and assessment of the rheumatologist. Since treatment success for therapy with bDMARDs is assessed within the first 6 months according to EULAR (European Alliance of Associations for Rheumatology) recommendations [17], 6 months were selected as the time horizon for prediction. The baseline visits of the t2t courses were categorized according to whether they were found to be effective or ineffective within the first 6 months of treatment.

Data were split into a training set (90% of the original dataset) and a test set (10% of the original dataset). To avoid data leakage between the two datasets, it was ensured that one patient was included in either the test-set or training-set. In addition, it was ensured that distributions of the therapy outcomes (ineffective or not) were similar among training and test set (stratified split). Iterative imputation, a method that predicts the missing variable as a function of other variables, was applied to input variables to handle missing data points. The hyperparameters, i.e., those parameters that are set before each training step, were optimized by using a model grid, with fixed hyperparameters (grid search).

The model grid contained 17 base models with different configurations described in the supplemental material in Table S3. We applied nested five-fold cross-validation on the training set, by iterating over an outer loop for model evaluation and iterating over an inner loop within each outer iteration step for hyperparameter tuning in order to avoid overfitting. Also, during the cross-validation process, split was performed group-wise, i.e., per patient.

Since the outcome distribution was highly imbalanced, we also incorporated different sampling strategies into the machine learning (ML) pipeline: synthetic minority over-sampling for numerical and categorical features (SMOTE-NC) of the minority class (“ineffective”) and random undersampling of the majority class (“effective”).

As a selection metric for the best model during nested-cross-validation, we collected the area under the receiver operating characteristic (AUC) for each medication, cross-validation-fold, test set, and sampling strategy, since AUC provides a generic metric to judge the overall model performance. The collection of model performance metrics per medication and model configuration can be found in the supplemental material in Table S3. The overall accuracy, i.e., the correctly predicted instances divided by all instances, was not evaluated, due to the imbalance of the dataset: Given a non-responder-rate of < 10%, a model that would always predict therapy response would still have a good (> 90%) accuracy, which could be misleading when evaluating the model-performance.

### Explainability

To evaluate the impact of the individual parameters on the outcome, we used the python library SHAP (“SHapley Additive exPlanations”), a game-theoretic approach for feature importance evaluation. In its original field, game theory, these numbers (“Shapley values”) reflect the contributions of a player in a coalition of players to the game-outcome. In machine learning, they reflect the contribution of a variable to the prediction model outcome [18]. Moreover, SHAP reflects interactions between variables and can reveal patterns via global explanations, by summarizing all local explanations of local predictions per instance.

All statistical analyses were conducted in python 3.9, using the python packages scikit-learn for machine learning, SHAP for feature importance analysis, and the tableone library for descriptive statistics [19].

## Results

Data from 1397 patients suffering from rheumatoid arthritis at the beginning of a treatment course with a new bDMARDs were extracted from the BioReg. Taking the exclusion criteria into account, the number of treatment courses amounted to 1843.

### Treat-to-target (T2T) course characteristics

In Table 1, the characteristics of the first visit of each t2t course (as instance to be predicted) are summarized and grouped by the target variable “Ineffective.” Overall, co-therapy with other DMARDs than methotrexate (MTX), glucocorticoid (GC)-co-therapy, previous therapy with aTNF, higher scores in visual analogue scale (VAS) namely VAS patient (VAS-Pat) or VAS physician (VAS-Ph), and higher values in disease activity (reflected by tender joint count/TJC and swollen joint count/SJC) were significantly more frequent in ineffective t2t courses.

Assessing the *p*-values per medication revealed a more differentiated picture as presented in Table 2. The following variables were associated with significantly higher risk of non-response depending on the medication: GC co-therapy for (adalimumab) ADA and (etanercept) ETA, VAS-Pat for all drugs except ADA, VAS-Ph for (abatacept) ABA and (tocilizumab) TOC, previous therapy with aTNF for (certolizumab) CERT and TOC, SJC for TOC, DAS-28-ESR for TOC.

**Table 2:**
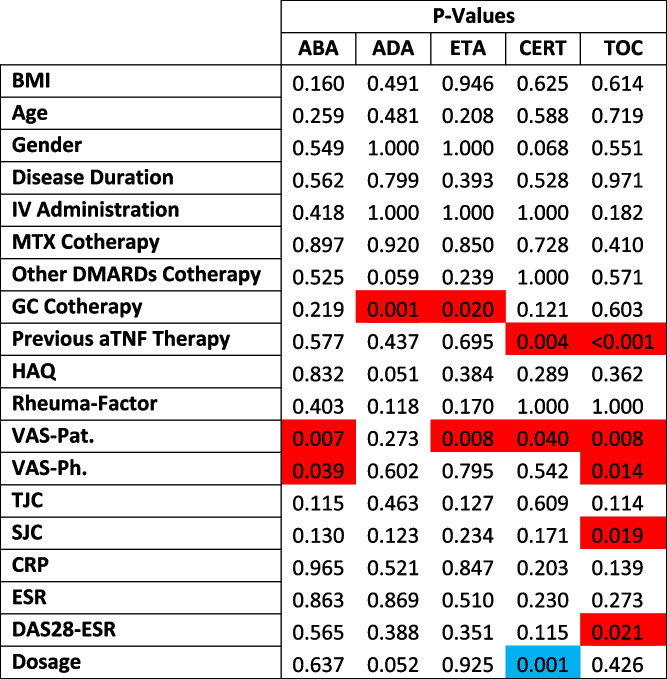
*P*-values grouped by drug. Factors with *p* < 0.05 and red color-code were associated with higher risk of non-response significantly. Only one factor (dosage) with *p* < 0.05 was associated with lower risk of non-response significantly. Dosage was normalized to mg/kg/day or mg/day depending on the medication

Higher dosage for CERT was associated with lower risk of ineffectiveness.

### Model quality metrics

**Fig. 2:**
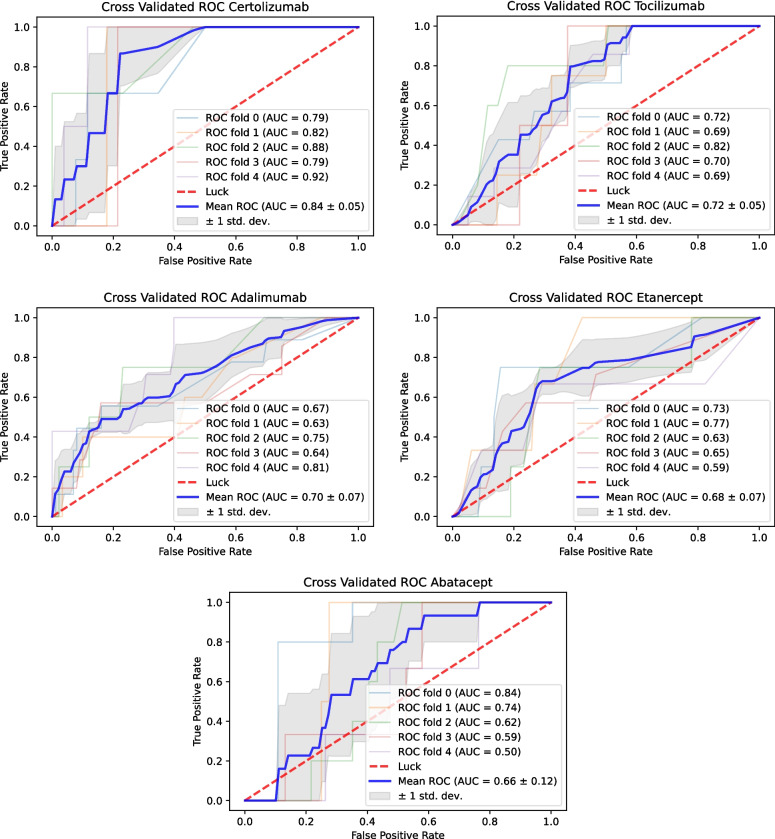
Area under the receiver operating characteristics for fivefold cross-validation

The area under the receiver operating characteristics for cross-validation per bDMARD could be calculated for ADA, ABA, CERT, ETA, and TOC (Fig. 2), ranging from 0.66 to 0.84. The model with the highest prognostic quality could be generated for CERT with an AUC of 0.84 (95% CI, 0.79–0.89). The most stable models with the lowest standard deviations (SD) over the 5 folds were generated for CERT with an AUC of 0.84 (SD: 0.05) and TOC with AUC of 0.72 (SD: 0.05).

Table 3 lists the models with the highest predictive quality and the associated strategy. Except for TOC, maximum AUC was achieved by addressing class imbalance: random undersampling combined with a Ridge classifier model achieved highest AUC for ABA, while the highest AUC for CERT was achieved by a combination of oversampling and a support vector classifier. For ADA, the best model performance was achieved by oversampling and XGBoost (extreme gradient boosting). For ETA, oversampling and random forest outperformed the other model and sampling combinations.

**Table 3:** Best models according to highest mean AUROC score per medication

Medication	Ineffective		Best model	Sampling strategy	Mean AUROC (95% CI)
	**No**	**Yes**			
**Abatacept**	212	20	Ridge classifier	RUS	0.66 (0.54–0.78)
**Adalimumab**	493	36	XG Boost	OVS	0.70 (0.68–0.74)
**Certolizumab**	150	11	SVC	OVS	0.84 (0.79–0.89)
**Etanercept**	530	23	RF Classifier	OVS	0.68 (0.55–0.87)
**Tocilizumab**	339	29	XG Boost	None	0.72 (0.69–0.77)

*XGBoost*, extreme gradient boosting; *SVC*, support vector classifier; *RF Classifier*, Random Forest Classifier; *RUS*, random undersampling; *OVS*, oversampling

### Variable importance

**Fig. 3:**
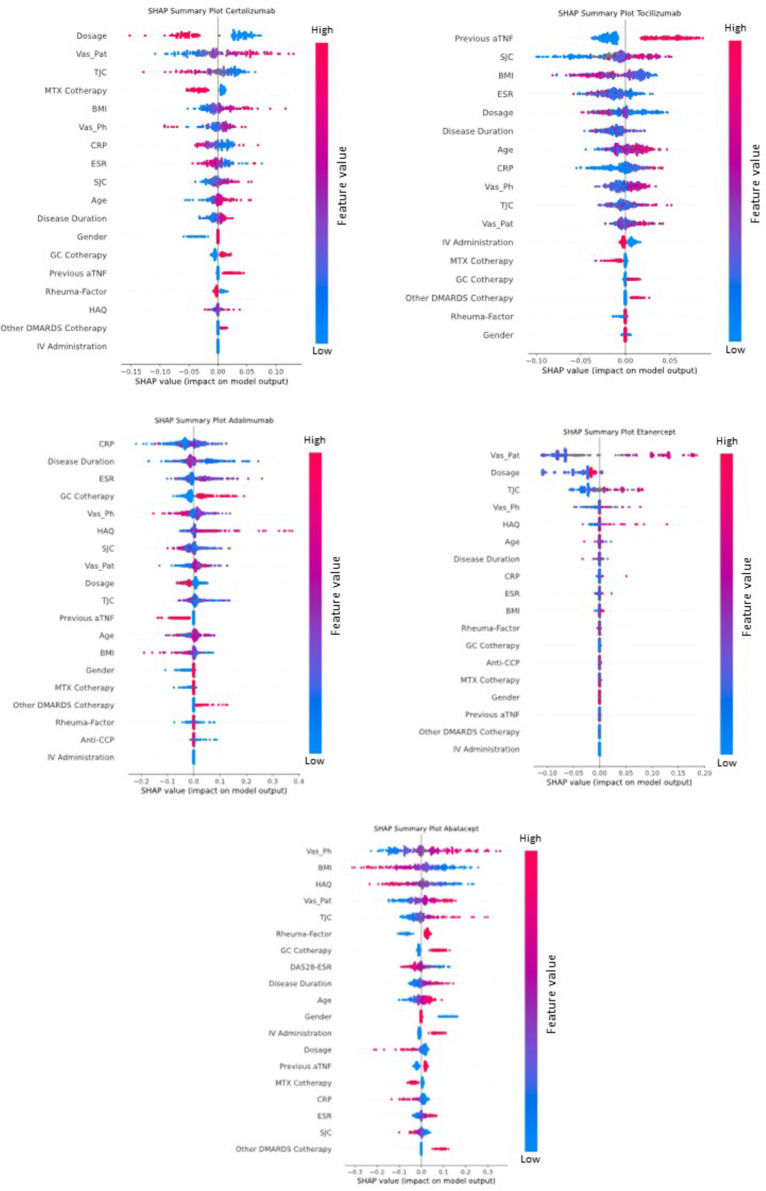
SHAP summary plots/impact of variables on model outcome. Variables are sorted in descending order of impact. Positive SHAP values indicate an effect in the direction of higher risk of ineffectiveness. Correspondingly, negative values indicate an effect of the factor in the direction of a lower risk for ineffectiveness. High values for the variables (features) are encoded in red; correspondingly low values are encoded in blue

The respective best performing models per bDMARD weighted the considered variables differently, as shown in the SHAP-summary plots in Fig. 3. A list of the most impactful variables encompassed different items or items in a different order for each individual bDMARD.

VAS scores were the common most predictive factor in abatacept (VAS-Ph) and etanercept (VAS-Pat). Co-therapy with GC had the highest impact on the ineffectiveness of adalimumab and VAS-Pat for certolizumab calculated by the SHAP explainer. The direction of VAS-Pat was identical for all bDMARDs, linking a higher feature level to a higher degree of ineffectiveness. In the case of CERT, a smaller dosage was linked to more probable ineffectiveness. Previous aTNF therapy was most predictive for ineffectiveness in case of TOC.

An interesting observation concerns the consistency of the direction of individual parameters across almost all bDMARDs. Whereas GC-co-therapy showed the same direction of effect with a higher GC dosage increasing the probability of ineffectiveness for all bDMARDs except for ETA, male gender was predictive not only for ineffectiveness with ABA but also for effectiveness with ADA. Likewise, a higher rheumatoid factor predicted ineffectiveness in ABA, whereas in CERT, a lower RF was linked to a worse clinical response (Fig. 3), although these observations were not statistically significant (Table 2).

## Discussion

In our study, we proved the feasibility of developing accurate machine learning models to predict with moderate to good prognostic quality the non-response of RA patients after 6 months in a real-world setting to *individual* bDMARDs. Furthermore, we could provide a quantification of each variable’s impact on the respective model per bDMARD using the explainable AI (XAI) framework SHAP.

The models in our studies yielded AUROC scores from 0.66 to 0.84 and consequently were considerably higher than the ones seen in the methodologically most similar studies [13, 14]. Herein, several machine learning models were applied to a Korean registry generating AUROC scores from 0.561 to 0.638 [13] and 0.511 to 0.694 [14] for the prediction of clinical response to bDMARDs in general. In our study, we used similar modeling techniques and furthermore addressed class imbalance by combining under- and oversampling techniques with different prediction models, which resulted in an improved model performance. Moreover, selecting drugs with more than 100 t2t courses and predicting missing data points by treating other features as input variables improved the training base and helped to build a robust model pipeline.

An important facet of our study is the characterization of feature importance including the direction of the respective feature importance on drug responsiveness. Although XAI methods are controversial regarding individual predictions (local explainability) [20], XAI methods can be used to explain how machine learning models work globally. Such global explanations can be combined with descriptive analysis to obtain insights on the importance of specific variables. In this respect, we found GC-co-therapy, VAS scores, and disease activity to be associated with higher risks of ineffectiveness in the whole cohort, regardless of the individual drug. Our findings are in line with the literature and add more detail, e.g., the significance of patient reported features, such as VAS patient (depicted in the SHAP Plots in Fig. 3) as important feature in all investigated bDMARDs as described in the study conducted by Lee et al. [13].

The importance of global assessments by patient and physician is reflected by the incorporation of these items into the different remission definitions based on the disease activity indices DAS28, SDAI, and CDAI. The central role of patients’ global assessment (PGA) was underscored in a report comparing CDAI and SDAI to the (most stringent) Boolean remission using data of 3 large clinical trials with adalimumab; the difference between CDAI and SDAI vs. Boolean remission was caused by higher patients’ VAS scores, leading to a redefined Boolean remission to allow a higher VAS score [21, 22].

In a recent paper by Capelusnik and Aletaha, the authors investigated predictors of response in three different large RCTs of aTNF including > 1300 patients after 30 weeks of treatment confirming the earlier notion of an inverse relationship of high baseline disease activity with a lower chance of achieving state targets (i.e., remission or low activity). In a more detailed analysis, PGA, among other values, was found significantly associated with a lower chance of response. Also, in our study, a higher PGA was predictive of a higher risk of bDMARD failure, which was significant in aTNF as well as in abatacept and tocilizumab. Also applying machine learning to predict response to DMARDs in RA established PGA to be an important predictor of remission in two recent reports [13, 23]. Duong et al. investigated predictors for methotrexate therapy and described a high PGA to be in the top 3 individual components predicting a poor response. As mentioned above, also in the Korean registry, patient-reported outcome, i.e., the PGA in RA, was revealed as the most important feature in the random forest as well as in the XGBoost model [13].

Remarkably, opposite effects of variables could also be observed, e.g., for gender and rheumatoid factor, although these effects did not reach statistical significance as demonstrated in Table 2.

The possible influence of gender/sex on drug responsiveness has come into focus in the last years. Besides proposed measures to adequately address this matter in future drug development [2], different drug retention rates and clinical effects have also been investigated in rheumatoid arthritis. This leads to the comprehension that women overall show a diminished response to drugs in rheumatoid arthritis [24]. Registry-derived data have demonstrated better responses or retention rates for male patients with rheumatoid arthritis to DMARDs in general and to aTNF specifically [12, 25–28]. This is in line with our findings, where gender was an important feature in all aTNF demonstrating a smaller risk of non-response for male patients especially in CERT and ADA. However, this was not statistically significant, only showing a statistical trend in CERT (*p* = 0.068).

Another feature of interest in the SHAP calculations was the presence of rheumatoid factor (RF), which lead to differential drug responsiveness depending on the specific bDMARD. Whereas a lower RF showed a trend to associate with a smaller risk of ineffectiveness in ABA and TOC, the opposite was seen in CERT, whereas the rest of the aTNF did not show a distinct direction of effect. The literature does not report consistent associations between the responsiveness to bDMARDs and RF. In a Taiwanese registry, overall RF positivity was associated with drug survival, which was statistically significant for ABA but not for aTNF and TOC, suggesting RF positivity as a biomarker for better responsiveness to abatacept [29]. An earlier systematic review and meta-analysis could not find such an association [30]. Conflicting data have also been published about the relationship of RF and aTNF treatment, although to our knowledge, differences between certolizumab and other aTNF have not been reported [31–34]. The different observation period, which was 6 months in our study opposed to one to several years in others, especially as the effect seen in the reported papers appeared after 6 months, may have contributed to partly discrepant findings in our study to previous reports [35, 36].

This study has some limitations. First, the models were developed using a single data source, the BioReg registry. Although BioReg includes data from hospital settings as well as private practices, a risk of systematic bias remains. As the prescription of a biological or targeted synthetic DMARD in Austria is mainly left to the discretion of the treating physician without the need to comply with objective outcome parameters used in clinical trials, our data might harbor known as well as unknown confounding variables, including confounding by indication. Moreover, the target variable “ineffectiveness” in the registry was set solely based on the opinion of the treating rheumatologist, which limits generalizability and comparability compared to studies, where specific thresholds for DAS-28-ESR or other objective measures were used to create a binary outcome variable. However, taking this approach often mirrors clinical practice. Furthermore, our study sample was small, mirroring a rather homogenous middle European population. The overall small sample size may explain why smoking status shows weak association with ineffectiveness as only 16 patients were past or current smokers and showed no treatment response, which is not in line with literature as smoking is consistently reported as having high association with treatment outcome.

Our described methodology should therefore be evaluated using independent datasets.

Embedding such models in a clinical setting to support treatment decisions raises the question of how an individual prediction should be presented to rheumatologists. A purely binary prediction with the result non-responder vs. responder would carry a high risk of misclassification, since, as can be seen in Fig. 2, a 100% sensitivity can never be achieved for the data examined, except for CERT and TOC, and this only if a high false positive rate is accepted. The representation of the continuous risk as well as the AUROC per drug and model would be preferable to a purely binary statement, which should be the subject of future studies. It is also important to emphasize that this study does not exclusively look at bDMARD naïve patients; however, this may be beneficial in a real-world scenario if such models would be embedded in a software-assistant, supporting rheumatologists in their day-to-day work.

## Conclusions

In conclusion, developing accurate machine learning models to identify patients with a high risk of non-response before therapy with bDMARDs is feasible. The algorithms used in our study should be applied to additional data sources including larger registries to refine our models and evaluate feature importance to support treatment decision in a clinical setting.

### Supplementary Information

**Additional file 1: Table S1.** Originally available variables from raw dataset potentially affecting treatment outcome, categorized by inclusion/exclusion criteria. **Table S2.** Correlation Heatmap for cleaned input dataset. SDAI and CDAI were excluded due to correlation > 0.8 with TJC, SJC and DAS28-ESR. **Table S3.** Model outcome depending on class imbalancing technique. Highest AUCs with a maximum difference between train mean AUC and held out set of 0.1 were selected for final model evaluation to ensure a robust and stable model.

## Data Availability

The data underlying this article cannot be shared publicly due to data privacy of individuals. The data will be shared on reasonable request to the corresponding author and the registry.

## Abbreviations

BioReg: Austrian Biologics Registry
ABA: Abatacept
ADA: Adalimumab
ADA-Boost: Adaptive Boosting
Anti-CCP: Anti-cyclic citrullinated peptide
aTNF: Anti-tumor necrosis factor
AUC: Area under the curve
AUROC: Area under the receiver operating characteristic
bDMARDs: Biologic disease-modifying antirheumatic drugs
CDAI: Clinical Disease Activity Index
CERT: Certolizumab
CI: Confidence interval
CRP: C-reactive protein
DAS28: Disease Activity Score 28
DMARDs: Disease-modifying antirheumatic drugs
ESR: Erythrocyte sedimentation rate
ETA: Etanercept
EULAR: European Alliance of Associations for Rheumatology
ExtraTrees: Extra Trees Classifier
GaussProc: Gaussian Process Classifier
GC: Glucocorticoid
HAQ: Health assessment questionnaire
IV administration: Intravenous administration
KOBIO: Korean College of Rheumatology Biologics and Targeted Therapy Registry
LDA: Linear discriminant analysis
MTX: Methotrexate
OVS: Oversampling
RA: Rheumatoid arthritis
RF: Rheumatoid factor
RF classifier: Random forest classifier
ROC: Receiver operating characteristic
RUS: Random undersampling
SD: Standard deviation
SDAI: Simplified disease activity index
SHAP: SHapley Additive exPlanations
SJC: Swollen joint count
SMOTE-NC: Synthetic minority over-sampling for numerical and categorical features
SVC: Support vector classifier
t2t: Treat-to-target
