# Supplementary material for: Prediction of ineffectiveness of biological drugs using machine learning and explainable AI methods: data from the Austrian Biological Registry BioReg
Source: Arthritis Res Ther. 2024 Feb 8;26:44. doi: 10.1186/s13075-024-03277-x (PMC10851538; doi:10.1186/s13075-024-03277-x)
Supplement: Supplementary file 1 — Additional file 1: Table S1. Originally available variables from raw dataset potentially affecting treatment outcome, categorized by inclusion/exclusion criteria. Table S2. Correlation Heatmap for cleaned input dataset. SDAI and CDAI were excluded due to correlation > 0.8 with TJC, SJC and DAS28-ESR. Table S3. Model outcome depending on class imbalancing technique. Highest AUCs with a maximum difference between train mean AUC and held out set of 0.1 were selected for final model evaluation to ensure a robust and stable model. [file 13075_2024_3277_MOESM1_ESM.docx]

**Supplemental Materials**

| **field** | **missing_rate_raw_data** | **exclusion_inclusion_criteria** |
| --- | --- | --- |
| Allergic | 1.00 | excluded due to high missing rate in raw data |
| Opp_Inf | 1.00 | excluded due to high missing rate in raw data |
| currentNSAIDScox | 1.00 | excluded due to high missing rate in raw data |
| vas_pat | 1.00 | excluded due to high missing rate in raw data |
| pastNSAIDScox | 1.00 | excluded due to high missing rate in raw data |
| vas_fatigue | 1.00 | excluded due to high missing rate in raw data |
| pastNSAIDS | 1.00 | excluded due to high missing rate in raw data |
| vas_pain | 1.00 | excluded due to high missing rate in raw data |
| currentNSAIDS | 1.00 | excluded due to high missing rate in raw data |
| GC_route | 1.00 | excluded due to high missing rate in raw data |
| ConcDMARD_tot | 1.00 | excluded due to high missing rate in raw data |
| MTX_delivery | 1.00 | excluded due to high missing rate in raw data |
| HCQ_dose | 0.99 | excluded due to high missing rate in raw data |
| SSZ_dose | 0.98 | excluded due to high missing rate in raw data |
| LEF_dose | 0.97 | excluded due to high missing rate in raw data |
| tsDMARD_schedule | 0.94 | excluded due to high missing rate in raw data |
| tsDMARD_dose | 0.94 | excluded due to high missing rate in raw data |
| GC_dose | 0.84 | excluded due to high missing rate in raw data |
| MTX_dose | 0.80 | excluded due to high missing rate in raw data |
| anti_ccp | 0.57 | included to check, if missing rate remains over 0.33 threshold per medication |
| sjc | 0.38 | included to check, if missing rate remains over 0.33 threshold per medication |
| haq | 0.38 | included to check, if missing rate remains over 0.33 threshold per medication |
| rheuma_factor | 0.35 | included to check, if missing rate remains over 0.33 threshold per medication |
| tjc | 0.33 | included due to high clinical relevance |
| vas_ph | 0.19 | included due to high clinical relevance |
| crp | 0.11 | included due to high clinical relevance |
| vas_pat | 0.11 | included due to high clinical relevance |
| esr | 0.09 | included due to high clinical relevance |
| delivery (administration) | 0.07 | included due to high clinical relevance |
| currentDose_total (mg) | 0.06 | included due to high clinical relevance |
| bDMARD_currentInterval | 0.06 | included due to high clinical relevance |
| bDMARD_currentDose (mg/kg | 0.06 | included due to high clinical relevance |
| previoustsDMARD | 0.00 | excluded expert decision |
| MTX cotherapy | 0.00 | included due to high clinical relevance |
| LEF (Leflunomide) | 0.00 | excluded expert decision |
| SSZ (Sulfasalazine) | 0.00 | excluded expert decision |
| HCQ (Hydroxychloroquine) | 0.00 | excluded expert decision |
| GC (Glucocorticoid) cotherapy | 0.00 | included due to high clinical relevance |
| Other_DMARDs cotherapy | 0.00 | included due to high clinical relevance |
| Comorb_cardiovascular | 0.00 | excluded expert decision |
| Comorb_lung | 0.00 | excluded expert decision |
| Comorb_infections | 0.00 | excluded expert decision |
| Comorb_malignancy | 0.00 | excluded expert decision |
| Comorb_metabolic | 0.00 | excluded expert decision |
| Comorb_neuropsy | 0.00 | excluded expert decision |
| previouscsDMARD | 0.00 | excluded expert decision |
| Prev_tsDMARD | 0.00 | excluded expert decision |
| Prev_csDMARD | 0.00 | excluded expert decision |
| smoker | 0.00 | included first, but later excluded due to weak  association with ineffectiveness (see table 1) |
| disease_duration | 0.00 | included due to high clinical relevance |
| bmi | 0.00 | included due to high clinical relevance |
| gender | 0.00 | included due to high clinical relevance |
| age | 0.00 | included due to high clinical relevance |
| Infection | 0.00 | excluded expert decision |
| brand_name | 0.00 | excluded expert decision |
| Prev_bDMARD | 0.00 | excluded expert decision |
| Prev_btsDMARD | 0.00 | excluded expert decision |
| previousOtherBio | 0.00 | excluded expert decision |
| previousABA | 0.00 | excluded expert decision |
| previous_atnf | 0.00 | included due to high clinical relevance |
| previousRIT | 0.00 | excluded expert decision |
| previousTCZ | 0.00 | excluded expert decision |

***Table S1:*** *Originally available variables from raw dataset potentially affecting treatment outcome, categorized by inclusion/exclusion criteria.*


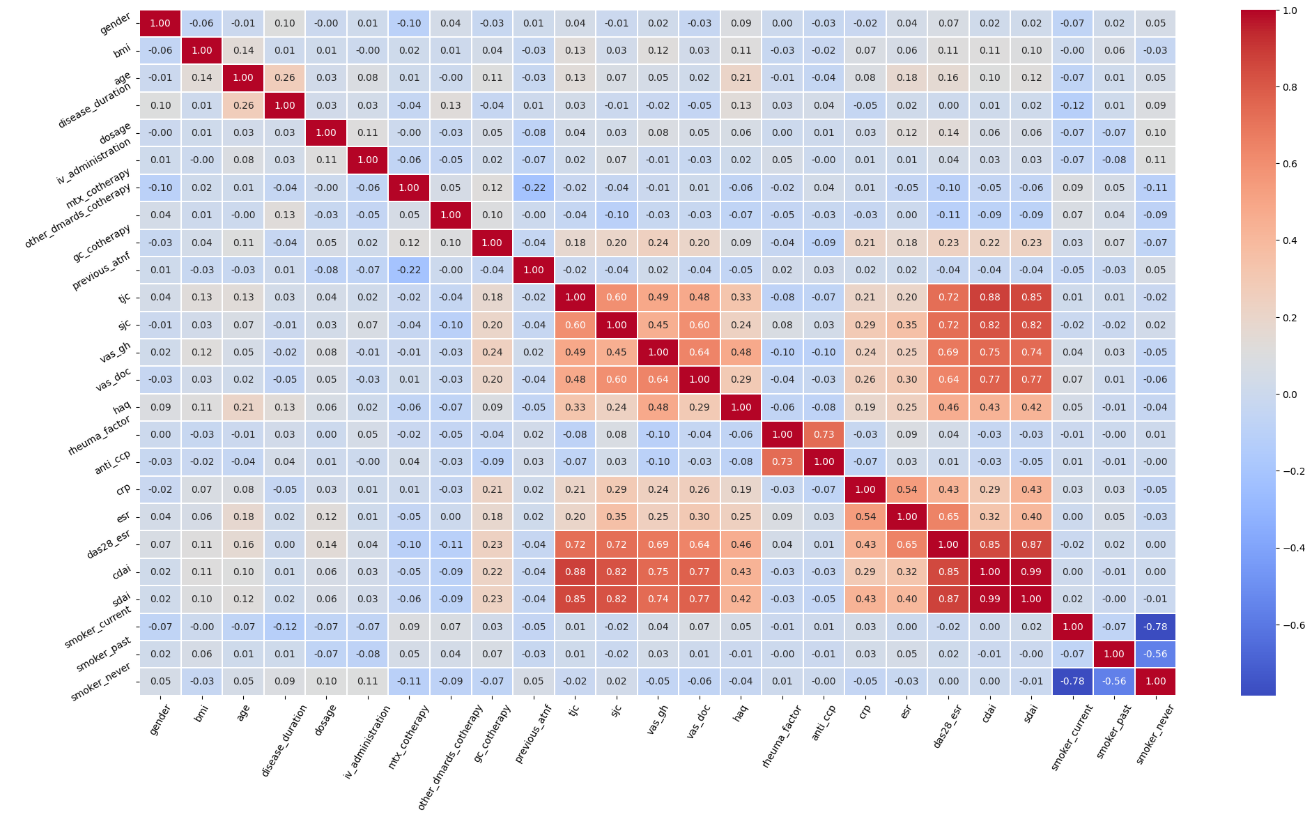


***Table S2:*** *Correlation Heatmap for cleaned input dataset. SDAI and CDAI were excluded due to correlation>0.8 with TJC, SJC and DAS28-ESR.*

| **medication** | **imb_strategy** | **algo** | **heldout_test_score** | **mean_test_score** | **std_test_score** |
| --- | --- | --- | --- | --- | --- |
| ETA | rus | LogReg | 0.68 | 0.51 | 0.14 |
| ETA | rus | Bayes | 0.61 | 0.45 | 0.1 |
| ETA | rus | LinSVM | 0.78 | 0.52 | 0.13 |
| ETA | rus | Ridge | 0.69 | 0.51 | 0.13 |
| ETA | rus | LDA | 0.56 | 0.49 | 0.1 |
| ETA | rus | DecTree | 0.19 | 0.55 | 0.18 |
| ETA | rus | k-NN | 0.37 | 0.55 | 0.14 |
| ETA | rus | SVC | 0.73 | 0.57 | 0.13 |
| ETA | rus | ANN | 0.08 | 0.36 | 0.15 |
| ETA | rus | GaussProc | 0.39 | 0.58 | 0.08 |
| ETA | rus | RndForest | 0.56 | 0.57 | 0.14 |
| ETA | rus | ExtraTrees | 0.35 | 0.52 | 0.12 |
| ETA | rus | AdaBoost | 0.28 | 0.48 | 0.18 |
| ETA | rus | xgBoost | 0.1 | 0.55 | 0.12 |
| ETA | rus | Stacking | 0.56 | 0.61 | 0.13 |
| ETA | rus | Voting | 0.66 | 0.52 | 0.1 |
| ETA | rus | SGD | 0.68 | 0.54 | 0.12 |
| ETA | ovs | LogReg | 0.82 | 0.5 | 0.04 |
| ETA | ovs | Bayes | 0.68 | 0.39 | 0.08 |
| ETA | ovs | LinSVM | 0.66 | 0.51 | 0.04 |
| ETA | ovs | Ridge | 0.7 | 0.5 | 0.16 |
| ETA | ovs | LDA | 0.58 | 0.44 | 0.12 |
| ETA | ovs | DecTree | 0.63 | 0.5 | 0.11 |
| ETA | ovs | k-NN | 0.47 | 0.52 | 0.05 |
| ETA | ovs | SVC | 0.35 | 0.51 | 0.19 |
| ETA | ovs | ANN | 0.44 | 0.34 | 0.1 |
| ETA | ovs | GaussProc | 0.49 | 0.66 | 0.12 |
| **ETA** | **ovs** | **RndForest** | **0.63** | **0.68** | **0.07** |
| ETA | ovs | ExtraTrees | 0.53 | 0.46 | 0.13 |
| ETA | ovs | AdaBoost | 0.59 | 0.44 | 0.23 |
| ETA | ovs | xgBoost | 0.29 | 0.49 | 0.25 |
| ETA | ovs | Stacking | 0.4 | 0.44 | 0.09 |
| ETA | ovs | Voting | 0.45 | 0.42 | 0.11 |
| ETA | ovs | SGD | 0.63 | 0.47 | 0.11 |
| ABA | rus | LogReg | 0.89 | 0.61 | 0.05 |
| ABA | rus | Bayes | 0.4 | 0.61 | 0.13 |
| ABA | rus | LinSVM | 0.59 | 0.59 | 0.08 |
| **ABA** | **rus** | **Ridge** | **0.6** | **0.66** | **0.12** |
| ABA | rus | LDA | 0.72 | 0.57 | 0.07 |
| ABA | rus | DecTree | 0.53 | 0.49 | 0.11 |
| ABA | rus | k-NN | 0.44 | 0.58 | 0.16 |
| ABA | rus | SVC | 0.9 | 0.56 | 0.1 |
| ABA | rus | ANN | 0.85 | 0.5 | 0.1 |
| ABA | rus | GaussProc | 0.57 | 0.59 | 0.15 |
| ABA | rus | RndForest | 0.52 | 0.59 | 0.14 |
| ABA | rus | ExtraTrees | 0.57 | 0.59 | 0.11 |
| ABA | rus | AdaBoost | 0.49 | 0.53 | 0.22 |
| ABA | rus | xgBoost | 0.53 | 0.55 | 0.1 |
| ABA | rus | Stacking | 0.68 | 0.64 | 0.05 |
| ABA | rus | Voting | 0.42 | 0.62 | 0.11 |
| ABA | rus | SGD | 0.48 | 0.59 | 0.11 |
| ABA | ovs | LogReg | 0.49 | 0.65 | 0.08 |
| ABA | ovs | Bayes | 0.52 | 0.59 | 0.1 |
| ABA | ovs | LinSVM | 0.36 | 0.53 | 0.17 |
| ABA | ovs | Ridge | 0.64 | 0.62 | 0.12 |
| ABA | ovs | LDA | 0.68 | 0.57 | 0.13 |
| ABA | ovs | DecTree | 0.32 | 0.45 | 0.11 |
| ABA | ovs | k-NN | 0.81 | 0.54 | 0.06 |
| ABA | ovs | SVC | 0.83 | 0.55 | 0.14 |
| ABA | ovs | ANN | 0.55 | 0.49 | 0.06 |
| ABA | ovs | GaussProc | 0.84 | 0.51 | 0.08 |
| ABA | ovs | RndForest | 0.91 | 0.58 | 0.14 |
| ABA | ovs | ExtraTrees | 0.8 | 0.59 | 0.06 |
| ABA | ovs | AdaBoost | 0.59 | 0.61 | 0.15 |
| ABA | ovs | xgBoost | 0.82 | 0.57 | 0.19 |
| ABA | ovs | Stacking | 0.77 | 0.46 | 0.14 |
| ABA | ovs | Voting | 0.84 | 0.58 | 0.11 |
| ABA | ovs | SGD | 0.57 | 0.53 | 0.09 |
| ADA | rus | LogReg | 0.54 | 0.59 | 0.13 |
| ADA | rus | Bayes | 0.46 | 0.63 | 0.12 |
| ADA | rus | LinSVM | 0.54 | 0.55 | 0.11 |
| ADA | rus | Ridge | 0.53 | 0.58 | 0.12 |
| ADA | rus | LDA | 0.46 | 0.64 | 0.12 |
| ADA | rus | DecTree | 0.78 | 0.57 | 0.07 |
| ADA | rus | k-NN | 0.64 | 0.63 | 0.07 |
| ADA | rus | SVC | 0.55 | 0.57 | 0.15 |
| ADA | rus | ANN | 0.6 | 0.5 | 0.07 |
| ADA | rus | GaussProc | 0.42 | 0.54 | 0.14 |
| ADA | rus | RndForest | 0.61 | 0.66 | 0.13 |
| ADA | rus | ExtraTrees | 0.34 | 0.66 | 0.1 |
| ADA | rus | AdaBoost | 0.59 | 0.53 | 0.16 |
| ADA | rus | xgBoost | 0.6 | 0.64 | 0.09 |
| ADA | rus | Stacking | 0.48 | 0.51 | 0.09 |
| ADA | rus | Voting | 0.51 | 0.66 | 0.11 |
| ADA | rus | SGD | 0.57 | 0.67 | 0.05 |
| ADA | ovs | LogReg | 0.52 | 0.66 | 0.1 |
| ADA | ovs | Bayes | 0.4 | 0.67 | 0.1 |
| ADA | ovs | LinSVM | 0.56 | 0.6 | 0.08 |
| ADA | ovs | Ridge | 0.48 | 0.68 | 0.09 |
| ADA | ovs | LDA | 0.47 | 0.68 | 0.12 |
| ADA | ovs | DecTree | 0.65 | 0.58 | 0.05 |
| ADA | ovs | k-NN | 0.51 | 0.59 | 0.1 |
| ADA | ovs | SVC | 0.56 | 0.45 | 0.08 |
| ADA | ovs | ANN | 0.52 | 0.57 | 0.11 |
| ADA | ovs | GaussProc | 0.56 | 0.65 | 0.07 |
| **ADA** | **ovs** | **xgBoost** | **0.73** | **0.7** | **0.07** |
| ADA | ovs | ExtraTrees | 0.48 | 0.71 | 0.1 |
| ADA | ovs | AdaBoost | 0.71 | 0.58 | 0.12 |
| ADA | ovs | xgBoost | 0.65 | 0.68 | 0.11 |
| ADA | ovs | Stacking | 0.53 | 0.67 | 0.05 |
| ADA | ovs | Voting | 0.42 | 0.69 | 0.05 |
| ADA | ovs | SGD | 0.45 | 0.54 | 0.13 |
| CERT | rus | LogReg | 0.75 | 0.68 | 0.16 |
| CERT | rus | Bayes | 0.69 | 0.66 | 0.15 |
| CERT | rus | LinSVM | 0.63 | 0.48 | 0.17 |
| CERT | rus | Ridge | 0.88 | 0.56 | 0.14 |
| CERT | rus | LDA | 0.75 | 0.6 | 0.13 |
| CERT | rus | DecTree | 0.22 | 0.64 | 0.11 |
| CERT | rus | k-NN | 0.88 | 0.59 | 0.21 |
| CERT | rus | SVC | 0.69 | 0.64 | 0.15 |
| CERT | rus | ANN | 0.44 | 0.38 | 0.17 |
| CERT | rus | GaussProc | 0.69 | 0.56 | 0.24 |
| CERT | rus | RndForest | 0.5 | 0.5 | 0,00 |
| CERT | rus | ExtraTrees | 0.5 | 0.5 | 0,00 |
| CERT | rus | AdaBoost | 0.56 | 0.62 | 0.1 |
| CERT | rus | xgBoost | 0.69 | 0.52 | 0.11 |
| CERT | rus | Stacking | 0.81 | 0.68 | 0.09 |
| CERT | rus | Voting | 0.88 | 0.71 | 0.13 |
| CERT | rus | SGD | 0.44 | 0.54 | 0.21 |
| CERT | ovs | LogReg | 0.47 | 0.65 | 0.17 |
| CERT | ovs | Bayes | 1,00 | 0.65 | 0.15 |
| CERT | ovs | LinSVM | 0.44 | 0.66 | 0.16 |
| CERT | ovs | Ridge | 0.19 | 0.62 | 0.22 |
| CERT | ovs | LDA | 0.56 | 0.65 | 0.19 |
| CERT | ovs | DecTree | 0.63 | 0.48 | 0.17 |
| CERT | ovs | k-NN | 0.38 | 0.53 | 0.16 |
| **CERT** | **ovs** | **SVC** | **0.88** | **0.84** | **0.05** |
| CERT | ovs | ANN | 0.19 | 0.39 | 0.26 |
| CERT | ovs | GaussProc | 0.66 | 0.63 | 0.08 |
| CERT | ovs | RndForest | 0.81 | 0.58 | 0.13 |
| CERT | ovs | ExtraTrees | 0.94 | 0.64 | 0.11 |
| CERT | ovs | AdaBoost | 0.81 | 0.62 | 0.14 |
| CERT | ovs | xgBoost | 0.63 | 0.53 | 0.2 |
| CERT | ovs | Stacking | 0.56 | 0.63 | 0.11 |
| CERT | ovs | Voting | 0.78 | 0.57 | 0.1 |
| CERT | ovs | SGD | 0.69 | 0.67 | 0.15 |
| TOC | rus | LogReg | 0.78 | 0.65 | 0.13 |
| TOC | rus | Bayes | 0.49 | 0.66 | 0.08 |
| TOC | rus | LinSVM | 0.73 | 0.64 | 0.14 |
| TOC | rus | Ridge | 0.67 | 0.64 | 0.14 |
| TOC | rus | LDA | 0.61 | 0.62 | 0.12 |
| TOC | rus | DecTree | 0.66 | 0.49 | 0.2 |
| TOC | rus | k-NN | 0.47 | 0.66 | 0.1 |
| TOC | rus | SVC | 0.7 | 0.66 | 0.13 |
| TOC | rus | ANN | 0.53 | 0.64 | 0.07 |
| TOC | rus | GaussProc | 0.56 | 0.73 | 0.08 |
| TOC | rus | RndForest | 0.74 | 0.66 | 0.14 |
| TOC | rus | ExtraTrees | 0.64 | 0.65 | 0.15 |
| TOC | rus | AdaBoost | 0.81 | 0.62 | 0.16 |
| TOC | rus | xgBoost | 0.73 | 0.66 | 0.17 |
| TOC | rus | Stacking | 0.61 | 0.6 | 0.2 |
| TOC | rus | Voting | 0.61 | 0.66 | 0.12 |
| TOC | rus | SGD | 0.7 | 0.68 | 0.1 |
| TOC | ovs | LogReg | 0.77 | 0.54 | 0.06 |
| TOC | ovs | Bayes | 0.69 | 0.57 | 0.14 |
| TOC | ovs | LinSVM | 0.75 | 0.54 | 0.04 |
| TOC | ovs | Ridge | 0.78 | 0.6 | 0.14 |
| TOC | ovs | LDA | 0.76 | 0.6 | 0.15 |
| TOC | ovs | DecTree | 0.67 | 0.6 | 0.15 |
| TOC | ovs | k-NN | 0.49 | 0.68 | 0.07 |
| TOC | ovs | SVC | 0.81 | 0.45 | 0.11 |
| TOC | ovs | ANN | 0.71 | 0.63 | 0.19 |
| TOC | ovs | GaussProc | 0.5 | 0.6 | 0.25 |
| TOC | ovs | RndForest | 0.77 | 0.62 | 0.2 |
| TOC | ovs | ExtraTrees | 0.74 | 0.66 | 0.17 |
| TOC | ovs | AdaBoost | 0.84 | 0.6 | 0.08 |
| TOC | ovs | xgBoost | 0.69 | 0.66 | 0.2 |
| TOC | ovs | Stacking | 0.58 | 0.73 | 0.09 |
| TOC | ovs | Voting | 0.66 | 0.65 | 0.17 |
| TOC | ovs | SGD | 0.75 | 0.54 | 0.08 |
| **TOC** | **no** | **xgBoost** | **0.7** | **0.72** | **0.05** |

***Table S3:*** *Model outcome depending on class imbalancing technique. Highest AUCs with a maximum difference between train mean AUC and held out set of 0.1 were selected for final model evaluation to ensure a robust and stable model.*
